# e-Monitoring of Asthma Therapy to Improve Compliance in children using a real-time medication monitoring system (RTMM): the e-MATIC study protocol

**DOI:** 10.1186/1472-6947-13-38

**Published:** 2013-03-21

**Authors:** Erwin C Vasbinder, Hettie M Janssens, Maureen P M H Rutten-van Mölken, Liset van Dijk, Brenda C M de Winter, Ruben C A de Groot, Arnold G Vulto, Patricia M L A van den Bemt

**Affiliations:** 1 Department of Hospital Pharmacy, Erasmus Medical Center, Nc-212, P.O. Box 2040, Rotterdam, CA 3000, The Netherlands; 2 Department of Hospital Pharmacy, Groene Hart Ziekenhuis, B1.43, Bleulandweg 10, Gouda, HH 2803, The Netherlands; 3 Department of Paediatrics, div. Paediatric Respiratory Medicine, Erasmus Medical Center/Sophia Childrens Hospital, Room Sp3456, P.O. Box 2060, Rotterdam, CB 3000, The Netherlands; 4 lnstitute for Medical Technology Assessment / Institute of Health Policy and Management, Erasmus University Rotterdam, P.O. Box 1738, Rotterdam, DR 3000, The Netherlands; 5 NIVEL, P.O. Box 1568, Utrecht, BN 3500, The Netherlands

**Keywords:** Real-time medication monitoring, Text-message reminders, Medication adherence, Inhaled corticosteroids, Children, Asthma

## Abstract

**Background:**

Many children with asthma do not have sufficient asthma control, which leads to increased healthcare costs and productivity loss of parents. One of the causative factors are adherence problems. Effective interventions improving medication adherence may therefore improve asthma control and reduce costs. A promising solution is sending real time text-messages via the mobile phone network, when a medicine is about to be forgotten. As the effect of real time text-messages in children with asthma is unknown, the primary aim of this study is to determine the effect of a Real Time Medication Monitoring system (RTMM) with text-messages on adherence to inhaled corticosteroids (ICS). The secondary objective is to study the effects of RTMM on asthma control, quality of life and cost-effectiveness of treatment.

**Methods:**

A multicenter, randomized controlled trial involving 220 children (4–11 years) using ICS for asthma. All children receive an RTMM-device for one year, which registers time and date of ICS doses. Children in the intervention group also receive tailored text-messages, sent only when a dose is at risk of omission. Primary outcome measure is the proportion of ICS dosages taken within the individually predefined time-interval. Secondary outcome measures include asthma control (monthly Asthma Control Tests), asthma exacerbations, healthcare use (collected from hospital records, patient reports and pharmacy record data), and disease-specific quality of life (PAQLQ questionnaire). Parental and children’s acceptance of RTMM is evaluated with online focus groups and patient questionnaires. An economic evaluation is performed adopting a societal perspective, including relevant healthcare costs and parental productivity loss. Furthermore, a decision-analytic model is developed in which different levels of adherence are associated with clinical and financial outcomes. Also, sensitivity analyses are carried out on different price levels for RTMM.

**Discussion:**

If RTMM with tailored text-message reminders proves to be effective, this technique can be used in daily practice, which would support children with suboptimal adherence in their asthma (self)management and in achieving better asthma control and better quality of life.

**Trial registration:**

Netherlands Trial Register NTR2583.

## Background

Asthma is the most common chronic childhood disease in industrialised countries and its prevalence has been increasing in the past years [1,2]. As in adults, asthma in children is associated with more hospitalisations, a decreased quality of life [3,4] and a substantial economic burden [5]. Children themselves report several negative consequences of asthma: feeling ill, limitations in peer interactions and medication annoyances [6]. Other problems include limited sports participation and school attendance [7]. These phenomena indicate that many children do not have sufficient asthma control, in spite of the availability of effective maintenance therapy in the form of inhalation corticosteroids (ICS). In a Dutch study 55% of the children with doctor-diagnosed asthma had insufficient control [8]. Poor adherence to ICS is an important risk factor for insufficient asthma control [9,10]. Studies show that adherence to ICS ranges from 40 to 70% [11-17].

The disruptive effect of non-adherence on asthma treatment implies that solutions are needed for improving adherence. Up to now, many interventions focus on education of parents and children. Review studies show that such educational interventions can result in a lower risk of hospital admissions but the effect on other outcomes is less clear [18,19]. A meta review on adherence showed that although education seems plausible for explaining adherence, the effects of educational interventions aimed to improve adherence were yet unclear [20]. A promising, yet complex, approach is to combine several interventions, e.g. improving the patient-doctor relationship, training the doctor’s communication skills and simplifying asthma medication [21].

Lately, information and communication technology (ICT)-solutions have been proposed to improve adherence and their effectiveness has been shown [22-24]. Examples are internet-based monitoring of asthma symptoms [25] and audiovisual reminding to take asthma medication [26]. Reminding patients through the sending of text-messages is a simple method with low intrusiveness and relatively low costs [27]. Text-message reminding might be especially suitable for unintentionally non-adherent patients, e.g. patients who forget to take their medication [28]. Several systematic reviews have shown that text-messaging is effective in the improvement of health outcomes or in changing health behaviour [24,29,30]. Examples include improved blood glucose levels in obese type 2 diabetes patients [31], higher level of physical activity [32], higher smoking cessation rates [33,34] and improved self-efficacy in young patients with diabetes type 1 [35]. In adult asthma patients positive results of daily text-message alerts have been reported as well: adherence to inhaler medication was 18% higher in patients receiving a 12 week intervention with text-message reminders [36].

A concern with repetitive sending of text-messages before every intake may be that patients get accustomed to receiving reminders leading to wearing-off of the adherence improving effect. To avoid this “alert-fatigue”, a more sophisticated approach is needed for optimal and enduring adherence improvement. Such an approach may consist of sending time-tailored text-message reminders that are sent only if a drug dose is at risk of omission. This technique needs the use of Real Time Medication Monitoring (RTMM), which is an adaptation of the Medication Event Monitoring System (MEMS). Like MEMS, RTMM uses an electronic medication dispenser that records the date and time the dispenser is opened. MEMS has proven to provide objective and reliable data of adherence and it has been used to measure medication adherence of various patient populations [37,38]. RTMM delivers the same type of data but, as opposed to MEMS, RTMM registers medication intake data in real time at a central data-server. This real time information is directly available, which enables sending text-messages to patients who are at risk of missing a dose of their medication.

The effect of sending time-tailored text-messages has not been studied extensively before. One randomized controlled trial for oral medication in adult diabetic patients has shown RTMM to be effective [39]. Also, preliminary results of a study in HIV-infected adults using RTMM show that patients receiving tailored text-message reminders improve adherence to antiretroviral therapy as well as rates of viral suppression [40]. The application of RTMM in children using inhalation medication has not been studied before and therefore needs further investigation. Since enhancement of inhalation therapy with RTMM is still an innovative and expensive technique, RTMM equipment and software are only available for research purposes. Before this technique can be further developed into a design suitable for regular care, more data are needed on cost-effectiveness and patient acceptance.

Therefore, in this study we investigate the impact of RTMM with time-tailored text-message reminding on adherence to ICS in children with asthma. Secondary aim is to determine the effect of RTMM on asthma-control. Finally, cost-effectiveness and patient acceptance of RTMM are studied.

## Methods/design

### Design

This study is a one year, multicenter, randomized controlled trial in children who use ICS for asthma. All children receive an RTMM-device which registers time and date of administered ICS doses. Children in the intervention group receive “time-tailored” text–messages that are only sent when a dose is at risk of omission. Patients in the control group do not receive such text-messages.

### Ethical approval

The medical ethics committee of the Erasmus Medical Center has approved the study protocol (protocol number MEC-2011-143, Netherlands Trial Registry code NTR2583, http://www.trialregister.nl).

Study data are coded in order to guarantee privacy of participants. Before entering the study, all participants are asked for written informed consent.

### Participants

Patients are recruited from five outpatient clinics in the Netherlands: St Lucas Andreas Hospital, Academic Medical Center, BovenIJ Hospital (all in Amsterdam), Erasmus MC (in Rotterdam) and Groene Hart Ziekenhuis (in Gouda). The inclusion criteria for participants are:

• *Age at start of the study is 4 to 11 years*. Children aged 12 years or older tend to show a more individual medication behaviour, with a smaller role for parents compared to younger children. Also, the Asthma Control Test, a questionnaire for measuring asthma control, was only validated for children aged 4 to11 years [41,42].

• *Doctor diagnosed asthma for at least six months*. This criterion aims to exclude patients with transient wheezing e.g. due to viral respiratory tract infections. Shortly after the diagnosis of asthma, patients may also be better motivated for treatment than in later stages of their disease. That is why we have chosen to aim for patients with chronic asthma. These are also the patients who are on maintenance therapy with ICS.

• *ICS use for at least three months*. In the first period of ICS use, adherence rates may be higher than normal. Therefore, only patients with chronic ICS use are included [10].

• *Use of a pressurized metered dose inhaler* (*pMDI*). The RTMM-devices used for electronic adherence measurement are only compatible with pMDIs. Children using ICS with nebulizers or dry powder inhalers can therefore not be included.

• *Use of fluticasone*, *fluticasone*/*salmeterol or beclomethasone*. The experimental RTMM-devices have been developed to accommodate only fluticasone (Flixotide^®^), fluticasone/salmeterol (Seretide^®^) or beclomethasone (QVAR^®^) inhalers. Children using other types of ICS can therefore not be included. Unless clinically indicated, childrens’ asthma medication will not be changed to fit this inclusion criterium.

• *At least one parent*/*caregiver has a mobile phone*. In the intervention group real-time text-message reminders are sent via the mobile telephone network. Also, alerts for low battery status of the RTMM-devices are automatically communicated with text-message reminders.

We aim to include 44 children per hospital into the study. From the hospital administrations of each participating hospital, records are randomly selected of children aged 4–11 years and diagnosed with asthma at least 6 months ago. After verification of the other inclusion criteria, a patient information leaflet is handed out or sent to the parents of the potential participants. Parents are contacted and invited to visit the paediatric outpatient department for an intake interview. In case of participation, at least one of the childs’ parents has to give written informed consent. If a hospital is unable to include the required number of patients, they will be recruited from one of the other participating hospitals.

Children are randomly assigned to the intervention group or the control group. We use block randomization per hospital with blocks of 16 patients. Initially, physicians, researchers, and patients are blinded for randomisation. However, randomisation is unblinded after start of the study period, when patients find out whether they receive text-message reminders or not.

**Figure 1:**
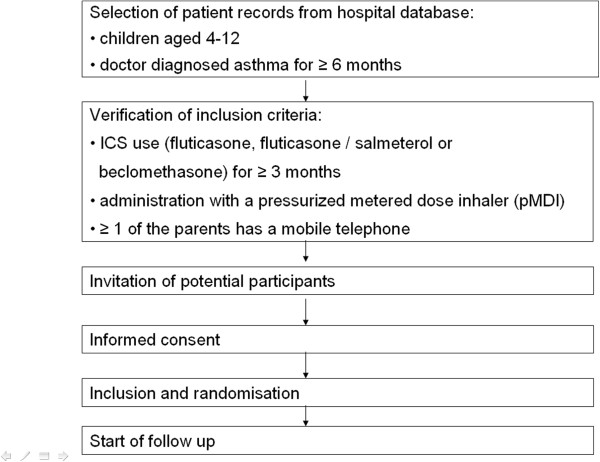
Patient section.

A flowchart of patient selection, randomisation and data collection is shown in Figure 1.

### Intervention

All children, both in the intervention and in the control group, receive an RTMM device for one year. ICS inhalations are registered by the RTMM-device which operates as follows: each time the pMDI is fired a data message containing patient-identification and time and date of administration is sent to the study database using the mobile telephone network. In order to prevent incomplete registration caused by insufficient network connection, the RTMM-device is designed to use two different networks: the mobile data-network and the regular mobile telephone network. If both networks are unavailable at the time of inhalation, a data message is prepared for sending at a later moment.

Only in the intervention group, time-tailored text-message reminders are sent to the parents and, if the child has a mobile phone, also to the child, in order to warn that a dose is at risk of being forgotten. Parents and children are not always together, for example if children are at school. As such, there may be a problem if the child has missed its dose and is already at school when the text-message is received by its parents. In that case, parents will not be able to verify if the child takes its medication. To ensure that text-messages are sent before the child goes to school (morning dose) or to bed (evening dose), a text-message is sent automatically if no ICS dose has been registered within 15 minutes after the planned time of inhalation. Such a short time interval may be less important when children are not at school, for example during weekends. For that reason, time intervals are individually defined (‘time-tailored’) for each patient for each day of the week. This is also thought to improve patient-acceptance of RTMM, reducing the so called “alert fatigue”.

Patients in the intervention group who live in an area with a very poor mobile network connection may occasionally receive an unnecessary text-message reminder if an ICS administration can not be reported to the RTMM-database in time. When the RTMM-network connection is re-established, the data message is sent. This phenomenon is closely monitored during the study period.

### Data collection

#### Outcome measures

The primary outcome measure is adherence to ICS, defined as the proportion of all prescribed dosages taken by the child within a six hour time-frame around the planned time of inhalation, i.e. from 3 hours before until 3 hours after. This is a common measure for twice daily dosing regimens [43-46]. In addition, we will look at other time-frames, missed doses and extra doses. These data are calculated from the RTMM data on ICS use, which are collected as described earlier in the “intervention”-section.

Secondary outcome measures are asthma control, frequency of asthma exacerbations, disease specific quality of life, healthcare use for asthma and school/work absence. These data are also used for calculation of the cost effectiveness of RTMM in children with asthma.

Asthma control is measured in several ways. The childhood Asthma Control Test (ACT) is filled out each month during the entire study in order to avoid seasonal differences [47]. The ACT is a questionnaire validated for children aged 4–11 years [41,42]. The ACT is a simple 7-item questionnaire, which has been shown to be useful in the detection of poorly controlled asthma. The ACT-score is calculated by adding the scores of all items; the ACT-score ranges from 0 to 27. The cut-off score is 19 points: 19 points or less means uncontrolled asthma, 20 points or more means controlled asthma. The ACT can also be used for measuring changes in asthma control. The minimally important difference between consecutive ACT scores is 3 points [48]: if two consecutive ACT-scores differ three points or more, the improvement or deterioration of asthma control is substantial and clinically relevant. In this study, asthma control is also measured using the frequency of asthma exacerbations and healthcare use. Pharmacy record data retrieved from the community pharmacy, are checked once at the end of the study period for high dose, short term oral corticosteroid use indicating asthma exacerbations. These pharmacy record data are also screened for new prescriptions of asthma medication, indicating a recent visit to a physician. Finally, the pharmacy record data are used to calculate medication costs.

Apart from screening pharmacy record data, healthcare use is also collected at the start and end of the study period by screening patient records and the hospital administration for visits to the outpatient clinic and for hospital admissions. Healthcare use and asthma related absence from school (child) or work (parent) are also assessed in a patient interview every 3 months. Asthma-specific quality of life is assessed by filling the standardized Paediatric Asthma Quality of Life Questionnaire (containing 23 items with a 7 point scale per item) (PAQLQ(S)) at the beginning and end of the study period [49]. The domains include activities, asthma symptoms and emotional function. The PAQLQ score ranges from 1 to 7 and is calculated as the average score of all items in a specific domain as well as an overall score. The PAQLQ can also be used to measure changes in quality of life. The minimally important difference in consecutive PAQLQ scores is 0.5. This is the minimum difference between two consecutive PAQLQ-scores that should be interpreted as a relevant improvement or deterioration of asthma-specific quality of life. A difference of 1 point indicates a moderate change and 1.5 is considered a large change [50].

#### Co-variables

We collect data on several factors that may be associated with adherence to medication, including age, gender, ethnicity (country of birth of child and parents), type of ICS (fluticasone, fluticasone/salmeterol or beclomethasone), ICS-dose, dosing frequency of ICS, type of asthma related co-medication (betasympathicomimetics, antihistaminergic agents, decongestives, antibiotics etc.), use of a spacer, parental level of education, parental Dutch language skills (assessed by investigators on a 5 point scale), smoking habits of parents (at home), family stability (child lives with both parents together / only with mother / only with father / both parents alternatively) [9], family income, professional occupation of parents, pets, mutations of asthma medication during the study period, existing spirometry data (from the past 3 months) and the occurrence of adverse events. Parental medication beliefs are measured using the Beliefs about Medicines Questionnaire Specific (BMQ Specific). This contains a scale for beliefs in the necessity of ICS and one for concerns about long term toxicity and disruptive effects of ICS. Both scales range from 5 to 25, with higher scores indicating stronger beliefs [51,52]. The data are collected from medical records at the beginning and end of the study period. In addition, at the beginning and end of the study period, and each 3 months in between, parents are interviewed by research assistants for collection of relevant data that cannot be extracted from medical records or questionnaires. Research assistants are trained by the research team before taking patient interviews.

#### Acceptance of RTMM

Since this study is an early evaluation of a medical innovation, we will pay special attention to the acceptance of RTMM using online focus groups (OFGs). These OFG discussions provide a convenient and comfortable way of joining group discussions and enable dialogue between participants who may not otherwise have spoken with each other. Discussions in computer-based focus groups produce the same quantity and quality of information obtained from face-to-face focus groups and are equally enjoyed by participants [53]. An additional advantage of OFGs is the larger contribution of less talkative participants in the discussion. The method also allows participants to join the discussion from their home and at a convenient time. OFGs are cost- and time-efficient due to the automatic and accurate capture of the discussion data. Children’s familiarity with the internet further pleads in favour of this methodology in our study. The OFGs are conducted following recently developed guidelines for online data collection [54].

In this study the OFGs are used to assess factors that would positively or negatively influence acceptance of RTMM and to capture more detailed information on how children and parents manage RTMM use. Eight children in the intervention group aged 9–11 years are asked to participate in an OFG. For younger children, eight parents are asked to participate in an OFG. Thus, two focus groups are created: one for children and one for parents. Participants are asked to respond anonymously to questions introduced by the researcher and to each others’ comments. Questions concern participants’ views on the usefulness and acceptability of specific components of the intervention. The researcher acts as moderator by regularly checking the postings and by asking additional questions to clarify participants’ views. The OFGs are carried out in the second half of the follow-up period. Table 1 provides a chronologic overview of all data that are collected in this study.

**Table 1:** Collection of outcome measures and co-variables in chronologic order

**Elapsed time since inclusion (months)**	**0**	**1**	**2**	**3**	**4**	**5**	**6**	**7**	**8**	**9**	**10**	**11**	**12**
Study visit/patient interview	X			X			X			X			X
Registry of patient characteristics	X												
Adherence to ICS (continuous Real Time Medication Monitoring, RTMM)	X	X	X	X	X	X	X	X	X	X	X	X	X
Asthma control in past month (Asthma Control Test, ACT)	X	X	X	X	X	X	X	X	X	X	X	X	X
Asthma control in past 3 months (collecting spirometry data)	X			X			X			X			X
Collecting data on healthcare use and school/work absence in the past 3 months				X			X			X			X
Collecting number of visits and admissions to hospital for asthma in the past 12 months	X												X
Screening public pharmacy dispensing data from the past 12 months (measuring asthma control, healthcare use and medication costs)													X
Medication Beliefs (Beliefs about Medicines Questionnaire,BMQ)	X												X
Asthma specific quality of life in past week (PAQLQ)	X												X
Patient acceptance of RTMM (Online Focus Groups, OFGs)								X	X	X	X	X	X

### Data monitoring

Data are initially collected on a case report form (CRF) and on questionnaires (hard copy). After each patient interview data are manually copied to a digital CRF. Data entry errors are minimized by using multiple choice options and fixed data fields. At the end of the study 10% of entered data are checked by a second person. If data entry errors are found, additional portions of 10% of the data are checked until no errors are found within a portion. Also, a periodic back-up of the study database of each hospital is made and checked for missing data. Access to the research databases is secured by passwords. Changing the format of the study documentation or study databases is restricted to the primary investigator. New versions are distributed from the central study location.

### Data analysis

The sample size calculation was based on the primary outcome measure: adherence to ICS. We use the adherence data from our observational study [15] in which adherence to ICS was electronically measured with RTMM in children (<12 years old) with asthma. In this dataset, 4 subgroups with different adherence patterns could be distinguished: patients with very poor adherence (≤5%), poor adherence (mean 34%), good adherence (mean 78%) and excellent adherence (≥95%). We assumed that patients with very poor adherence would not show any relevant improvement, since it is likely that they deliberately stopped taking ICS. The adherence rate in this group is not likely to be improved by the text-message intervention. The group with excellent adherence is also not expected to show improvement, since adherence is already nearly 100%. Adherence in both intermediate groups (poor, good adherence) is expected to improve by 10-15%. This estimated effect size was based on an adherence improvement reported in a systematic review on the effect of (non-tailored) reminder systems on patient adherence to treatment [55]. Since the time-tailored text-message reminders used in our study are considered potentially more effective, we estimated the improvement at 15%.

Using these assumptions, we have simulated the adherence data of the control and treatment groups. Since the four adherence subgroups cannot be analyzed in one single regression model, we used a mixture of regressions (“mixture model”) in order to assess the effect of the intervention. We also calculated levels of statistical power at different group sizes. Requiring a power of at least 0.8 and assuming an adherence improvement of 15%, we calculated that a group size of 110 per arm is needed to detect the expected difference. Data analysis is based on an intention-to-treat principle. All patients with a follow-up of at least three months, regardless of whether they actually finish the intervention, are included in the analysis. The two groups are compared for baseline characteristics. Co-variables that may influence adherence levels, and therefore may confound the effect of the text-message intervention on adherence, are added to the multivariable model. In addition, a sensitivity analysis is carried out using a per-protocol approach. In this analysis the effect is studies of patients who complete less than three months of follow-up and of patients who appear to have stopped using ICS early in follow-up (less than 1% of total prescribed inhalations are administered during the complete follow-up). Data are analysed with SPSS for Windows.

For calculation of the cost-effectiveness a prospective economic evaluation from a societal perspective is performed alongside the clinical trial. The one-year costs of all relevant health care utilization are included as well the direct non-healthcare costs and the costs of productivity losses when parent stay home to take care of their children. Costs will be related to adherence, asthma control and asthma-specific quality of life to calculate the following incremental cost-effectiveness ratios (ICERs):

1. Costs per 10% improvement in adherence

2. Costs per additional patient with minimal clinically important improvement in asthma control

3. Costs per additional patient with the minimal clinically important improvement in asthma quality of life. The uncertainty around the ICERs will be displayed on cost effectiveness-planes and cost-effectiveness acceptability curves.

Subsequently, a decision-analytic model is developed that includes different levels or forms of adherence and the outcomes, both clinical and costs, attributed to each level or form of adherence as well as different price levels for RTMM. For the base-case, this model is filled with estimates of the relationship between adherence on the one hand and asthma control, symptoms, exacerbations, quality of life and healthcare utilization on the other hand. These estimates are obtained from the clinical trial, where potential associations between adherence and outcomes are studied. This model is used to run extensive one-way and multivariate sensitivity analyses to simulate the anticipated benefits of improved adherence in terms of health outcomes and costs.

## Discussion

We designed a randomised controlled trial in children aged 4 to 11 using inhaled corticosteroids (ICS) for asthma. We will investigate the clinical and cost effectiveness of an intervention with Real Time Medication Monitoring (RTMM) with text-message reminders. Medication taking behaviour is monitored on a real-time basis, enabling immediate patient feedback through “time-tailored” text-message reminders that are only sent if the ICS is at risk of omission.

In this study, RTMM with text-message reminders is used as an adherence improving intervention. Three categories of adherence-enhancing strategies have been defined: enabling, consequence and stimulant [56]. Enabling strategies arm patients with the tools necessary for adherence, e.g. patient education and simplified medication regimens. Consequence strategies aim to reinforce adherence by providing incentives for acceptable adherence, e.g. instructing patients to maintain records of pill-taking or having patients monitor blood pressure at home. Stimulant strategies are aimed at prompting dose-taking. The RTMM with text-message reminders used in this study, is a stimulant strategy and therefore primarily targets unintentional non-adherence, e.g. forgetting to take a dose. This could limit the expected effect of our intervention, since adherence to ICS may also be influenced by intentional factors, like illness perceptions (perceived susceptibility and severity of the disease), the perceived benefits of treatment and theoretical barriers to treatment (e.g. concerns about (potential) side effects) [57]. However, RTMM with text-message reminders may also diminish intentional non-adherence by providing patients with feedback, while appealing to a desire to appear adherent when use is scrutinized by an outside party [55]. Receiving information that an inhalation is about to be missed, may also enable patients to adjust their medication taking behaviour, thus improving self-efficacy and asthma related quality of life [58].

### Strengths and limitations

A strength of this study is the use of RTMM as an objective and reliable method for adherence measurement. This method provides minimal room for bias, e.g. by socially acceptable patient response (patient self-report), misjudgement of patient behaviour (adherence questionnaires) and overestimation of adherence based on pharmacy refill data (refill rate, persistence) [13,59,60]. The RTMM device has been designed as a small add-on to the ICS inhaler. Since it does not need to be carried separately, it provides a patient-friendly way of measuring and stimulating adherence to ICS. This multi-centre study is the first to investigate RTMM with text-message reminders in a large sample of children with asthma. It is also the first to study the cost-effectiveness of RTMM in asthma. More data on cost-effectiveness are needed since the costs of this innovative technique are still substantial (approximately €750,= per patient per year) and are keeping physicians from using it in daily clinical practice. Health insurance companies also require more data on cost-effectiveness before covering costs for applying RTMM in asthma therapy.

Although electronic monitoring such as Real Time Medication Monitoring is considered more sensitive for measuring non-adherence than other, subjective tools for adherence measurement [13,61,62] the actual adherence to ICS may still be overestimated. All participating patients are aware that they are being observed, so they may act more adherent than in average daily practice. A common critique of electronic medication monitoring based on the time and date the inhaler is fired, is that it cannot be confirmed that the medication is actually taken. Only drug assays can confirm ingestion. However, studies comparing the sequence of medication events with projected and periodically measured concentrations of the drug in plasma, confirmed the validity of medication event monitors. Mismatches between medication events and actual dosing were too rare to create substantial differences between projected and actual concentrations of the drug in plasma [63-66]. Another concern with adherence measurement of ICS is the fact that registered doses may not have been administered correctly due to poor inhalation technique. This may have a negative influence on the effectiveness of ICS therapy [67] and therefore on asthma related quality of life and on patients’ motivation to adhere to therapy. This phenomenon is considered evenly distributed within intervention and control group, so we expect that the effect on the outcome measures adherence to ICS and “asthma control” is limited. A potential limitation of this study is the high quantity of outcome-measures and co-variables (Table 1). This may provoke partial non-response, leading to missing data. Another concern is the fact that both children using fluticasone and those using a combination of fluticasone and the long acting beta-agonist salmeterol are included into this study. It is well known that co-inhalation of a long acting beta-agonist causes bronchodilatation resulting in a relief from asthmatic symptoms. This may be rewarding for the asthma patient, possibly resulting in a better adherence. Besides, patients needing a combination of fluticasone and salmeterol may have more severe asthma than those who’s symptoms can be sufficiently controlled by ICS alone and may therefore be better motivated to adhere to their asthma therapy. To overcome this limitation we collect data on the type of ICS (fluticasone or fluticasone/salmeterol) as a co-variable, which enables us to include it as a confounder in the multi-variable analysis or to perform stratified analysis.

One of the inclusion criteria of this study is the use of ICS for at least three months. This is verified by checking medical records and by asking potential participants which drugs are used for asthma. This procedure, however, does not account for patients who have stopped using ICS without consulting a physician. If a part of the patients that are not included for not using an ICS still had an indication for taking ICS, they have a 0% adherence rate. Patients who, on the other hand, are included into the study, but in fact already have stopped using ICS, also have a 0% adherence rate. Since these phenomena are expected to be equally distributed among patients in the intervention and control group, the only potentially relevant effect is a decrease in statistical power. In order to quantify the effect of the latter (patients, who stopped using ICS but still enter the study) a sensitivity analysis is carried out in which the patients who took less than 1% of prescribed doses are excluded.

It is expected that RTMM has most value in patients with therapy resistant poor asthma control. In daily practice, it is often unclear whether the prescribed asthma treatment is suboptimal (e.g. dose is too low, inconvenient inhaler) or the treatment is adequate, but the patient does not adhere to it. In the current study, however, we have chosen not to make a pre-selection of patients with poor asthma control or (suspected) non-adherence. Instead, asthma control is measured during the entire follow up, which enables us to investigate if poor asthma control at baseline is associated with response to the RTMM intervention.

It is crucial for correct sending of text-messages and for correct adherence measurement that any changes in mobile telephone (used for receiving text-message reminders), ICS dose, ICS dosing frequency and type of ICS, is correct at any moment during the study period. To ensure this, patients are requested to inform the investigators about any relevant changes. In addition these data are verified in the patient interview each three months of the study period. If RTMM-devices are detected that have not been actuated for more than a month, patients are contacted once to check for technical failures. This intervention is documented.

The trial-based cost-effectiveness analysis proposed here aims to explicitly estimate the cost-effectiveness of RTMM. However, at this early stage of development of RTMM with text-message alerting, adherence in stead of asthma control was used as the primary outcome measure. Therefore, the trial may not allow definite conclusions on the impact of this intervention on the cost of asthma treatment. Hence, we will apply appropriate decision-analytic modelling techniques to simulate the anticipated benefits of improved adherence. Such a model needs to relate the different levels of exposure to ICS to levels of asthma control. A model like that allows extensive sensitivity analyses on both clinical outcomes and costs that are attributed to each level of adherence.

## Conclusion

RTMM with text-message reminders has the potential to support non-adherent patients in improving their asthma (self)management and in achieving better asthma control and better quality of life. RTMM could also provide physicians with the right information to treat patients who have poorly controlled asthma despite ICS therapy. Additional evidence on the (cost) effectiveness of this innovative adherence improving strategy would contribute to making it available for use in daily clinical practice.

## Competing interests

The authors declare that they have no competing interests, other than mentioned under ‘Acknowledgements and funding’.

## Authors’ contributions

EV (coordinating investigator) and BW (study monitor) coordinated the study start-up and data collection in the participating hospitals. EV also participated in obtaining the GlaxoSmithKline grant and managed the medical ethics approval. HJ, MR, LD and AV and RG participated in the study design and in the e-MATIC study group. PB (primary investigator) designed the study, initiated the ZonMw and GSK grant request and supervises the multicenter study. MR designed and carries responsibility for the cost-effectiveness part of the study. EV and PB wrote the manuscript; all other co-authors commented on previous versions of the manuscript and agreed with the final content. The study was supervised by the e-MATIC study group, which participated in every phase of the study. The e-MATIC study group was responsible for supervising the study design, collection of data and writing of the manuscript. All authors read and approved the final manuscript.

## Authors’ information

e-Matic Study group

● N. Dahhan, Academic Medical Center/Emma Children’s Hospital, Department of Paediatrics, Amsterdam, The Netherlands

● B.H.M. Wolf, St. Lucas Andreas Hospital, Department of Paediatrics, Amsterdam, The Netherlands

● K.E. van Boven, Academic Medical Center/ Emma Children’s Hospital, Department of Paediatrics, Amsterdam, The Netherlands

● E.I.M. Blankman, BovenIJ Hospital, Department of Paediatrics, Amsterdam, The Netherlands

● M. Veenstra, Groene Hart Ziekenhuis and Zorg Brug, Department of Paediatrics, Gouda, The Netherlands

● F.G.A. Versteegh, Groene Hart Ziekenhuis, Department of Paediatrics, Gouda, The Netherlands.

## Pre-publication history

The pre-publication history for this paper can be accessed here:

http://www.biomedcentral.com/1472-6947/13/38/prepub
